# The Encyclopedia of Animal Nutrition (2nd Edition)

**DOI:** 10.5713/ab.24.0001B

**Published:** 2024-08-22

**Authors:** Yoo Yong Kim

**Affiliations:** 1 DCollege of Agriculture and Life Sciences, Seoul National University, Seoul 08826, Korea

CABI's 2nd edition of The Encyclopedia of Animal Nutrition is a valuable resource for anyone interested in animal nutrition.

This edition includes several significant features. It was written by a team of internationally renowned professionals in the field. It covers a wide range of animals, from agricultural animals to other animal species encountered in our daily lives, such as fish, honey bees, and others. It also addresses various subjects, including physiology, biochemistry, veterinary medicine, and feed sciences. As a result, this book can serve as a resource for both researchers and students of animal science, as well as a great reference book for anyone working in allied subjects such as veterinary medicine, zoology, food science, and human nutritional science.

This book is organized alphabetically, allowing readers, particularly graduate and undergraduate students, to readily look up specific terms in animal and human nutrition. Unlike traditional animal nutrition or biochemistry textbooks, this book includes thorough explanations of the definitions of key terminology, related metabolic processes and connected terms within the body. It also includes references to the source of each term's explanation, as well as recommendations for further reading in other books or papers.

Readers with some baseline knowledge in animal or human nutrition can use this book as a nutrition-specific encyclopedia, providing considerably more detailed and practical information than a general encyclopedia. The book is also intended to be extremely useful to the general public, enabling people to quickly find answers to nutrition-related concerns in their everyday lives.

